# Chronic kidney disease in a giant panda (*Ailuropoda melanoleuca*): a case report

**DOI:** 10.1186/s12917-023-03663-8

**Published:** 2023-08-23

**Authors:** Lingling Chang, Xiangyang L. Wang, Chenfei Yu, Chen-Hsuan Liu, Qiang Zhang, Yaping Wu, Ruoyi Jia, Qingyi Ma, Guanglin Pan, Dewen Tong, Xinglong Wang

**Affiliations:** 1 College of Veterinary Medicine, Northwest A&F University, Yangling, China; 2 Graduate Institute of Molecular and Comparative Pathobiology, School of Veterinary Medicine, National Taiwan University, Taipei, Taiwan; 3 Qinling Giant Panda Research Center, Xi’an, China

**Keywords:** Giant panda, Chronic kidney disease, Heart failure, Hypertension

## Abstract

**Background:**

Chronic kidney disease (CKD) is a common cause of morbidity and mortality in captive wildlife species. However, CKD has been rarely documented in giant pandas.

**Case presentation:**

The following report describes a case of an eight-year-old female giant panda showing clinical signs of epistaxis, bloody diarrhea, polyuria, azotemia and anemia. The animal died despite of supportive treatments. Necropsy was performed. Grossly, both kidneys were shrunken and scarred with pallor. Subcutis edema and petechia on the epicardium of the heart were observed. The tissue samples were made into paraffin sections and stained by H.E and special staining including Periodic Acid-Schiff (PAS), von Kossa, Masson’s trichrome, Phosphotungstic acid-hematoxylin (PTAH), and Congo red. Histopathology examination revealed severe chronic tubulointerstitial nephritis with marked interstitial fibrosis, glomerulosclerosis, tubular atrophy and calcification in kidneys, and acute necrotizing hemorrhagic myocarditis with calcification in heart. Other lesions included intestinal hemorrhage, hepatic fatty degeneration and necrosis with hemosiderin, and splenic hemosiderin.

**Conclusions:**

In summary, chronic kidney disease was finally diagnosed based on the association of clinical, gross, and histopathological findings. Heart failure secondary to CKD is the leading cause of death in this giant panda. The potential cause of CKD in this animal is possibly due to long term and uncontrolled hypertension. Blood pressure monitoring is essential in establishing the diagnosis and management of hypertension in giant panda.

## Background

The giant panda (*Ailuropoda melanoleuca*) is precious creature unique to China and is a global symbol of wildlife conservation. In spite of the great deal of public and scientific interest in giant pandas, diseases threatening to this species are scantly reported [1–4]. Chronic kidney disease (CKD) is a complex metabolic disease which implies irreversible and progressive loss of function and/or structure of the kidney. This progressing deterioration of the renal injury leads to the accumulation of toxins, causing uremia and, eventually, the death of animals [5]. Previous case reports showed that CKD was a common cause of morbidity and mortality in many captive wildlife species such as tigers and bears [6, 7]. The pathologic findings of nonspecific tubulointerstitial inflammation, fibrosis, and mineralization in the kidneys are pathogonomonic for CKD [5]. In giant pandas, CKD has been rarely documented, and the etiology and pathology of CKD in this species are usually undetermined [8]. This report described CKD occurred in a captive giant panda.

## Case presentation

The eight-year-old female giant panda was born in a nature reserve in Shaanxi Province, China. According to keepers, the giant panda has been thin since birth, but no abnormalities were found during routine medical checks. At the beginning of May 2022, the giant panda was presented to Qinling Giant Panda Research Center (Xi’an city, China) for a history of acute onset of unilateral nostril bleeding. Approximately two weeks later, the animal began to develop lethargy, anorexia and bloody diarrhea. Serum chemistry revealed marked azotemia with elevations in both creatinine (23.53 mg/dL, reference range 0.87–1.92 mg/dL) and urea nitrogen (112.01 mg/dL, reference range 5.32–27.17 mg/dL), and marked elevations in liver enzymes including aspartate aminotransferase (AST,1078 U/L, reference range 53-140.5 U/L) and alanine aminotransferase (ALT, 442 U/L, reference range 54–98 U/L). There were electrolyte abnormalities of hypochloridemia, hyponatremia, and hypocalcemia with markedly decreased levels of chloridion (20.31 mEq/L, reference range 94.92-105.69 mEq/L), sodion (118.0 mEq/L, reference range 129–135 mEq/L) and calcium (4.49 mg/dL, reference range 8.28–9.28 mg/dL). Hematology examination revealed anemia and severe thrombocytopenia with low levels of red blood cell count (3.47 × 10^12^/L, reference range 5.66–7.31 × 10^12^/L), white blood cell count (4.2 × 10^9^/L, reference range 9.0-15.2 × 10^9^/L), hematocrit (19.30%, reference range 30.3–40.9%), hemoglobin (71 g/L, reference range 107–146 g/L), and marked decrease of platelet count (13 × 10^9^/L, reference range 158–281 × 10^9^/L).Urinary sediment revealed that the parameters of urinary protein and occult blood were both positive suggestive of proteinuria and hematuria. Five days prior to death, the giant panda were treated with interferon α, arrestin and nutrient solution. Antibiotics of Ceftiofur sodium and Omeprazole were administrated for one day. Despite of supportive therapies, the clinical condition of the giant panda quickly deteriorated and progressed to oronasal bleeding, vomiting, polyuria and dyspnea. The giant panda finally died on 11 June 2022.

At necropsy, subcutaneous edema was observed. Both kidneys were shrunken and scarred with pallor. In the heart, focal extensive petechiae was seen on the epicardium. The tissue speciments from the kidneys, heart, lungs, spleen, liver, and small intestine were fixed in 10% neutral buffered formalin, embedded in paraffin and sectioned at 5 μm for routine H.E staining and microscopic examination. Additionally, the kidney tissue samples were specially stained with H.E, PAS and Masson’s trichrome in 2 μm sections, and von Kossa and Congo red in 5 μm sections. The heart tissue samples were specially stained by von Kossa and PTAH staining.

**Fig. 1:**
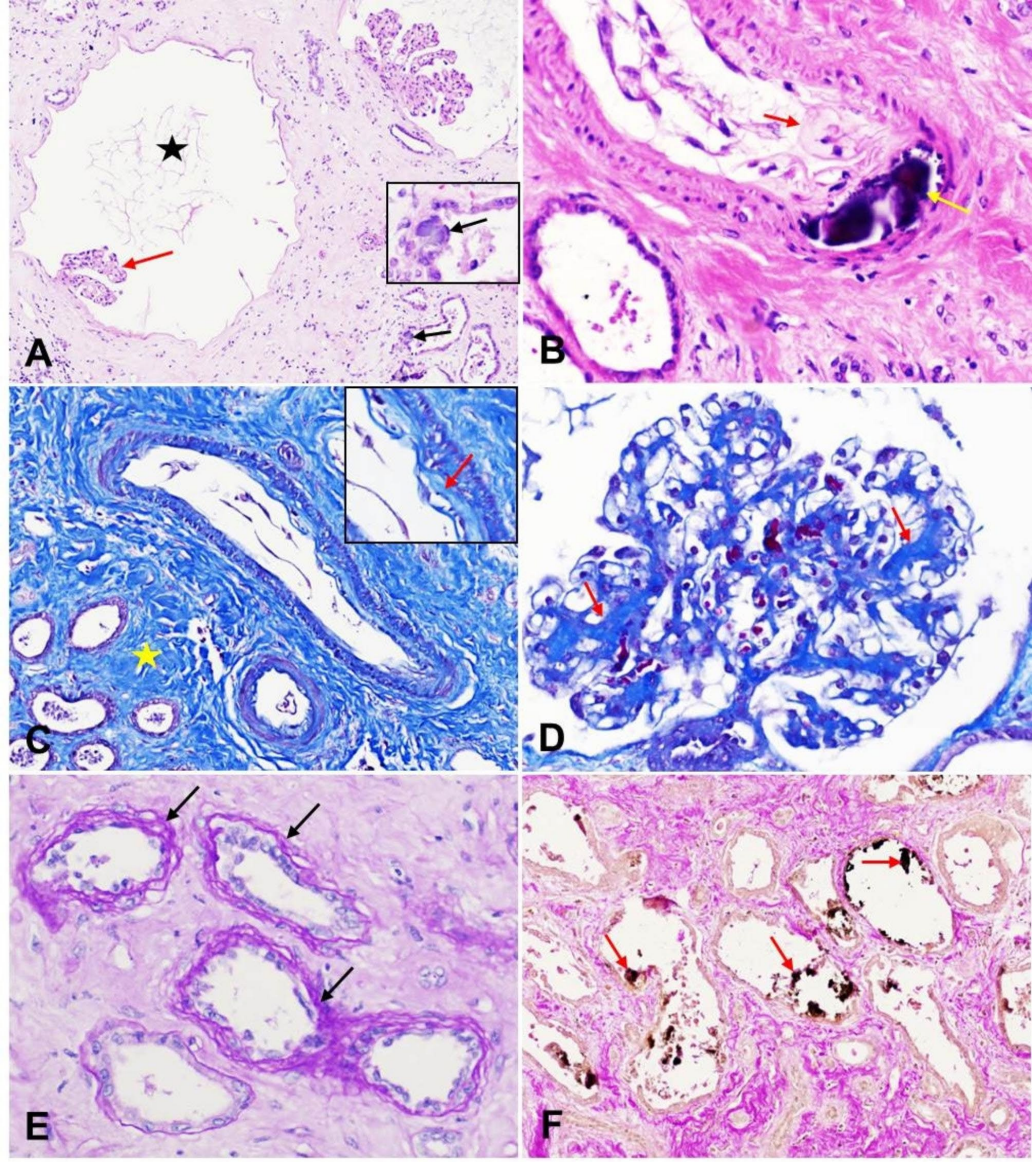
Histopathological changes in the kidney. **A**: One glomerulus had markedly ectatic Bowman’s space (star) with atrophic glomerular tufts (red arrow). Note the basophilic calcium deposition in the walls of renal tubules (black arrow). H.E staining, 200× magnification. **B**: The arterial wall was focally expanded by amorphous material of the intima (red arrow) (hyaline degenaration) and basophilic calcium deposition of media (yellow arrow). H.E staining, 400× magnification. **C**: A Masson’s trichrome staining demonstrated marked collagen depositon in the interstitium and arterial intima (arrow). Masson’s trichrome staining, 400× magnification. **D**: A Masson’s trichrome demonstrated collagen depositon in the glomeruli (arrow). Masson’s trichrome staining, 200× magnification. **E**: A PAS staining revealed markedly thickened and wrinkled (arrow) tubular basement membranes (peritubular fibrosis). PAS staining, 400× magnification. **F**: A von Kossa staining demonstrated the crystals within the renal tubular lumen were calcium deposition (arrow), which appeared basophilic crystals on H.E sections. von Kossa staining, 200× magnification

Histopathologically, in both kidneys, there was marked interstitial fibrosis which separated and replaced approximately 50% of tubules and glomeruli. Glomeruli showed diffuse cystic changes with capillary tufts atrophy (Fig. 1A) and Bowman’s capsules were moderately thickened.Tubules had moderately to severely thickened basement membranes. The tubular epithelium showed moderate to severe necrosis, desquamation, or atrophy. Within tubules, there were many proteinaceous fluid, occasional refractile basophilic crystals (Fig. 1A). Small numbers of lymphocytes infiltrated in the interstitium. Occasionally, there was mild deposition of amorphous material of the intima (hyaline degenaration) and calcification of media in the artery or arteriole (Fig. 1B). The Masson’s trichrome staining identified marked collagen deposition in the intima of renal artery (arteriolosclerosis) (Fig. 1C) and in the glomeruli (glomerulosclerosis) (Fig. 1D). The PAS staining demonstrated that the tubules had markedly thickened and wrinkled tubular basement membranes (peritubular fibrosis) (Fig. 1E). The von Kossa staining demonstrated the calcium deposition in the renal tubules(Fig. 1F), which appeared basophilic crystals on H.E sections. Congo red staining for amyloid on renal tissue sections was negative.

**Fig. 2:**
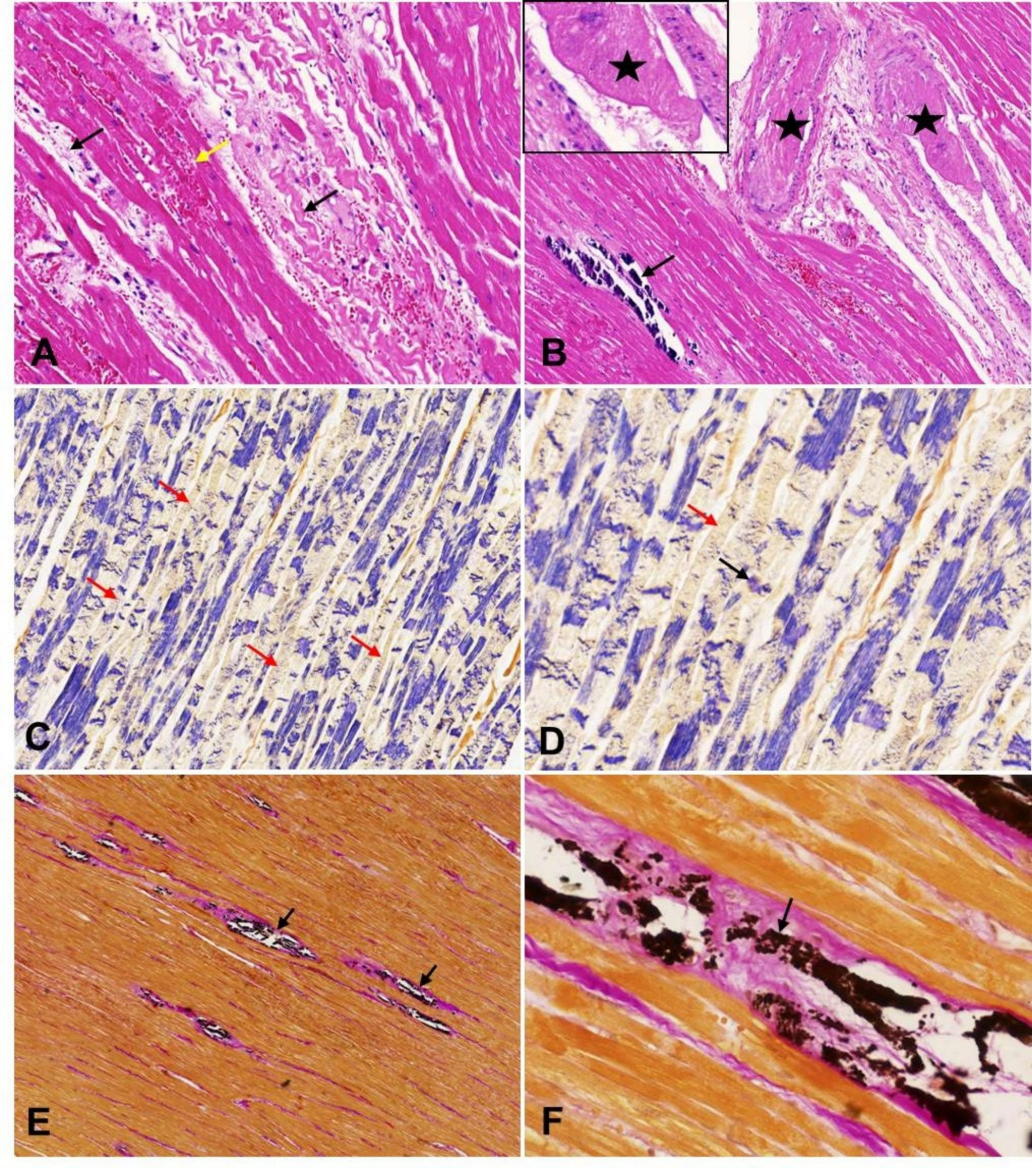
Histopathological changes in the heart. **A**: The myocardium showed focal coalescing necrosis (black arrow) and hemorrhage (yellow arrow). The necrotic cardiomyocytes were shrunken or fragmented (black arrow). H.E staining, 200× magnification. **B**: There was thrombi in the lumen of large vessels (star). Basophilic calcium deposition was noted in the myocardium (arrow). H.E staining, 200× magnification. **C**: A PTAH staining demonstrated marked myocardial necrosis. The necrotic myocardial fibers were stained yellowish brown, and the texture in the sarcoplasm was blurred or disappeared (arrow). PTAH staining, 200× magnification. **D**: High magnification of myocardial fiber necrosis. The cross striations in the sarcoplasm disappeared (red arrow) or exhibited blue mass (black arrow). PTAH staining, 200× magnification. **E**: A von Kossa staining demonstrated calcium deposition in the myocardium (arrow). von Kossa staining, 100× magnification. **F**: High magnification of calcium deposition in the myocardium (arrow). von Kossa staining, 400× magnification

In the heart, the myocardium had multifocal to coalescing areas of necrosis and hemorrhage (Fig. 2A). There was multiple coalescing foci of calcium deposition in the blood vessels and myocardium. Additionally, thrombi was noted in the lumen of large vessels (Fig. 2B). The necrotic myofibers manifested abrupt interruption and fragmentation of sarcoplasm, which had a much better visualization using the PTAH staining (Fig. 2C-D). Von Kossa staining demonstrated vascular and myocardial calcification (Fig. 2E-F).

In the small intestine (the segment is unkown), there was diffuse loss of the mucosal epithelial lining and moderate hemorrhage in the lamina propria. Vascular thrombosis was occasionally observed in the muscular layer. In the liver, there were multifocal foci of acute coagulative necrosis and extensive fatty degeneration particularly of centrilobular to midzonal hepatocytes. Hemosiderin in the Kupffer cells was frequently observed. In the spleen, white pulp was not prominent. Diffuse hemosiderin deposition was seen in the red purple. There were rare megakaryocytes scattered throughout the splenic parenchyma (extramedullary hematopoiesis).In the lung, multifocal anthracosis was observed around bronchioles and blood vessels.

## Discussion

Chronic kidney disease (CKD), also called chronic renal failure or chronic renal insufficiency, is a relentlessly progressive disease. This disease has been widely described in humans and animals. CKD causes accumulation of the waste compounds, which are normally removed or regulated by the kidneys, in the blood that results in CKD-associated complications [9, 10]. Clinically, the animals with CKD are characterized by a combination of signs of lethargy, anorexia, polydipsia, polyuria, vomiting, diarrhea, and weakness. Additionally, high blood pressure (hypertension), nasal bleeding, gastrointestinal tract bleeding, bone fractures are also frequently observed [5, 11]. These clinical manifestations are commonly observed in all causes of CKD and similar within different animal species. Due to these CKD signs are lack of specificity and may be seen in other disease, veterinarians will most often turn to blood tests and urine analysis to evaluate the kidney function. Clinicopathological parameters frequently associated with CKD include azotemia, proteinuria, metabolic acidosis, electrolyte abnormalities, anemia, and may progress to uremia [12]. For the present case, the clinical signs of epistaxis, bloody diarrhea, polyuria, and vomiting, blood results of azotemia, anemia, hypochloridemia, hyponatremia, and hypocalcemia, and urinalysis of proteinuria and hematuria are highly consistent with CKD. Histopathologically, severe interstitial fibrosis with glomerulus sclerosis, tubular atrophy, and calcification in the kidneys provide a definitive evidence for the diagnosis of CKD.The cause of CKD is poorly understood. The development of this disease is likely to be influenced by genetic, environmental, and individual patient factors. Generally, infectious and non-infectious etiologies have to be taken into consideration. In animals, the common causes of CKD include congenital renal malformation, chronic infection, hypertension, immune diseases, and acute kidney disease [11]. In this study, viral metagenome sequencing results showed that there was no evidence of the involvement of infectious virus (data not shown). Urinalysis revealed no bacterial, parasitic or fungal infection in the kidneys (data not shown). We speculated that the CKD in this giant panda was possibly caused by hypertension.

CKD is a common cause of hypertension in animals and contributed to the systemic hypertension. CKD can be both a cause and effect of hypertension, complicating the pathogenesis in individual cases [13]. In this giant panda, despite blood measurement was not performed, histopathologic lesions associated with hypertension in the kidney including arteriolosclerosis, vascular calcification, and glomerulosclerosis were identified [5]. Notably, arteriolosclerosis and vascular calcification play a vital role for the development of hypertension and CKD [14]. The calcium deposited within vessel walls can reduce vascular elasticity and contribute to the generation of vascular stiffness and hypertension [14–18]. Vascular calcifications could further exacerbate CKD [19].

The most notably clinical complication in animals with hypertension is bleeding due to vascular injury secondary to persistent systemic hypertension and CKD [13]. In this case, the initial clinical sign of epistaxis exhibited one month prior to death was possibly associated with hypertension. Epistaxis secondary to hypertension had been described in a giant panda with CKD [8]. In fact, several other sites of hemorrhage were identified histologically in this panda’s liver and spleen. These had deposition of hemosiderin and exhibited an end-stage response to chronic vascular damage as a result of persistent systemic hypertension. Additionally, hepertensive hemorrhage of ocular retina and central nervous system has also been described in animals [20, 21].

Hypertension not only contributes to the progression of CKD, but also the occurrence of cardiovascular disease including myocardial infarction, heart failure, and sudden death, which is the leading cause of mortality in patients with CKD [22]. In the heart, the valve and myocardium get calcified as well as the vessel walls. Myocardial calcification is a rare cause of severe heart failure [23]. For the present case, the heart showed vascular and myocardial calcification and severe myocardial necrosis, which contribute to heart failure and eventual death in this giant panda.

## Conclusion

In summary, chronic kidney disease was finally diagnosed based on the association of clinical, gross, and histopathological findings. Cardiovascular disease secondary to CKD is the leading cause of death in this giant panda. The potential cause of CKD in this animal is possibly due to long term and uncontrolled hypertension. Blood pressure monitoring is essential in establishing the diagnosis and management of hypertension in giant panda.

## Data Availability

All the histological images supporting the results in the current study is contained within the manuscript, and available from the corresponding author on reasonable request.
