# Correction: Chronic kidney disease in a giant panda (*Ailuropoda melanoleuca*): a case report

**DOI:** 10.1186/s12917-023-03731-z

**Published:** 2023-09-18

**Authors:** Lingling Chang, Xiangyang L. Wang, Chenfei Yu, Chen-Hsuan Liu, Qiang Zhang, Yaping Wu, Ruoyi Jia, Qingyi Ma, Guanglin Pan, Dewen Tong, Xinglong Wang

**Affiliations:** 1 College of Veterinary Medicine, Northwest A&F University, Yangling, China; 2 Graduate Institute of Molecular and Comparative Pathobiology, School of Veterinary Medicine, National Taiwan University, Taipei, Taiwan; 3 Qinling Giant Panda Research Center, Xi’an, China

**Correction: ***BMC Vet Res ***19**, **131 (2023)**

10.1186/s12917-023-03663-8

Following publication of the original article [1], the authors identified an error in one of the author names in the author list. The author name “Xiangyang L Wang” is wrong and should be corrected to “Xiangyang Wang”.
